# The Role of Mcl-1 in Embryonic Neural Precursor Cell Apoptosis

**DOI:** 10.3389/fcell.2021.659531

**Published:** 2021-04-20

**Authors:** Robert T. Flemmer, Sarah P. Connolly, Brittany A. Geizer, Joseph T. Opferman, Jacqueline L. Vanderluit

**Affiliations:** ^1^Division of BioMedical Sciences, Memorial University, St. John’s, NL, Canada; ^2^Department of Cellular and Molecular Biology, St. Jude Children’s Research Hospital, Memphis, TN, United States

**Keywords:** Bcl-2, neurogenesis, nervous system, development, cell death, Bax

## Abstract

Myeloid cell leukemia-1 (Mcl-1), an anti-apoptotic Bcl-2 protein, regulates neural precursor cell (NPC) survival in both the developing and adult mammalian nervous system. It is unclear when during the neurogenic period Mcl-1 becomes necessary for NPC survival and whether Bax is the sole pro-apoptotic target of Mcl-1. To address these questions, we used the nervous system-specific Nestin-Cre Mcl-1 conditional knockout mouse line (Mcl-1 CKO) to assess the anti-apoptotic role of Mcl-1 in developmental neurogenesis. Loss of Mcl-1 resulted in a wave of apoptosis beginning in the brainstem and cervical spinal cord at embryonic day 9.5 (E9.5) and in the forebrain at E10.5. Apoptosis was first observed ventrally in each region and spread dorsally over time. Within the spinal cord, apoptosis also spread in a rostral to caudal direction following the path of differentiation. Breeding the Mcl-1 CKO mouse with the Bax null mouse rescued the majority of NPC from apoptosis except in the dorsomedial brainstem and ventral thoracic spinal cord where only 50% were rescued. This demonstrates that Mcl-1 promotes NPC survival primarily by inhibiting the activation of Bax, but that Bax is not the sole pro-apoptotic target of Mcl-1 during embryonic neurogenesis. Interestingly, although co-deletion of Bax rescued the majority of NPC apoptosis, it resulted in embryonic lethality at E13, whereas conditional deletion of both Mcl-1 and Bax rescued embryonic lethality. In summary, this study demonstrates the widespread dependency on Mcl-1 during nervous system development.

## Introduction

Nervous system development follows a highly coordinated process. As formation of the neural tube completes, neurogenesis begins. Neural stem cells switch from dividing symmetrically to expand the neural stem cell pool to dividing asymmetrically and initiating neurogenesis by a stem cell and a committed neural progenitor cell. The number of neural precursor cells (NPC) and ultimately the number of neurons in the mature nervous system is regulated by a balance between survival- versus death- promoting proteins. The B cell lymphoma 2 (Bcl-2) family of pro- and anti-apoptotic proteins regulate this balance. Two anti-apoptotic Bcl-2 proteins, Myeloid cell leukemia-1 (Mcl-1) and Bcl-2 related gene long isoform (Bcl-xL) are required for cell survival during the process of neurogenesis. Mcl-1 is required for neural progenitor survival during the initial stages of neurogenesis, whereas Bcl-xL is required for the survival of newly generated neurons (Motoyama et al., 1995; Arbour et al., 2008; Fogarty et al., 2019). Nervous system-specific conditional deletion of both Mcl-1 and Bcl-xL results in apoptotic death of the entire CNS and lethality at embryonic day 12 (E12) indicating a requirement for both proteins throughout the developing nervous system (Fogarty et al., 2019). Conditional deletion of Bcl-xL alone, however, affects the survival of only select neuron populations including upper layer cortical neurons, cholinergic neurons, spinal cord interneurons, and motor neurons (Savitt et al., 2005; Fogarty et al., 2016; Nakamura et al., 2016). Less is known about whether Mcl-1 is required ubiquitously in NPC throughout the developing nervous system.

Mcl-1 is unique from the anti-apoptotic Bcl-2 proteins. Mcl-1 expression is tightly regulated, and Mcl-1 protein has a short half-life of only a few hours (Warr et al., 2005; Zhong et al., 2005; Adams and Cooper, 2007). Mcl-1 was initially linked to a potential role in cell differentiation as early studies showed that Mcl-1 expression increases as lymphoid cells differentiate (Kozopas et al., 1993). Germline Mcl-1 knockout mice are lethal at E3.5 due to a failure in trophoblast differentiation (Rinkenberger et al., 2000). Mcl-1 expression fluctuates during the cell cycle with expression peaking in M-phase as cells exit to differentiate and then decreasing thereafter (Harley et al., 2010). Furthermore, overexpression of Mcl-1 in embryonic NPC induces premature cell cycle exit (Hasan et al., 2013). Taken together, these studies suggest a potential role for Mcl-1 in cell cycle exit or differentiation.

In the developing nervous system, Mcl-1 is required for cell survival through the period of neurogenesis as neural stem cells differentiate into immature neurons (Fogarty et al., 2019). In nervous-system specific Mcl-1 conditional knockout mice, apoptosis is observed in proliferating Nestin+ neural precursor cells, as well as doublecortin + neuroblasts and βIII tubulin + immature neurons (Arbour et al., 2008). Although Mcl-1 conditional knockout mice are embryonic lethal at E15.5, it’s unclear when Mcl-1 becomes necessary for NPC survival. Mcl-1 expression begins early in nervous system development, with *mcl-1* mRNA detected at E10 (Fogarty et al., 2019). In the Mcl-1 CKO, apoptosis is observed as early as E10 in the developing spinal cord and by E11 in the forebrain demonstrating that Mcl-1 is required early in neurogenesis (Fogarty et al., 2019). As earlier time points have not been examined, it is unclear whether Mcl-1 is required prior to the onset of neurogenesis in the Nestin-positive neural precursor pool.

Here we used the nervous system specific Mcl-1 conditional knockout mouse to investigate the role of endogenous Mcl-1 in early nervous system development.

## Materials and Methods

### Mice

All mouse lines were maintained on a C57Bl/6J background. Mcl-1 conditional knockout (Mcl-1 CKO) mice were generated as previously described by breeding our Nestin:Cre mouse line (Berube et al., 2005) with the Mcl-1 floxed mouse line (Opferman et al., 2003; Arbour et al., 2008; Hasan et al., 2013; Fogarty et al., 2019). The Mcl-1 CKO/BaxNull embryos were generated by crossing the Mcl-1 CKO mouse line with the BaxNull mouse line (cat# 002994, Jackson Laboratories, MN, United States) (Knudson et al., 1995). The Mcl-1 CKO/BaxNull/BaxFloxed embryos were generated by breeding the Mcl-1 CKO/BaxNull line with the BaxFloxed mouse line (cat# 006329, Jackson Laboratories, MN, United States) (Takeuchi et al., 2005). Tissue samples were collected from each embryo during dissection for genotyping. DNA extraction was performed with the REDExtract-N-Amp^TM^ Tissue PCR Kit according to manufacturer’s instructions (Sigma-Aldrich Co., LLC, XNAT-100RXN, MO, United States). PCRs were performed with previously published primers for *cre*, *mcl-1* (Arbour et al., 2008; Malone et al., 2012), the null *bax* allele (Knudson et al., 1995) and the floxed *bax* allele (Takeuchi et al., 2005). Mice were housed on a 12-h light/dark cycle with access to food and water *ad libitum*. For timed pregnancies, mice were bred for 3 days and checked twice daily for vaginal plugs. The presence of a vaginal plug was taken as embryonic day 0.5. All experiments were approved by Memorial University of Newfoundland’s Institutional Animal Care Committee according to the Guidelines of the Canadian Council on Animal Care.

### Tissue Processing, Immunohistochemistry, and Western Blot

Pregnant dams were euthanized with an intraperitoneal injection of 300 μl of sodium pentobarbital (Euthanyl, 240 mg/ml, CDMV, QC, and CA) followed by cervical dislocation. Post-euthanasia, embryos were removed and fixed in 4% paraformaldehyde in phosphate buffered saline (1 × PBS), pH 7.4 overnight at 4°C. Embryonic tissues were cryoprotected in a 30% w/v sucrose solution in 1 × PBS then embedded in Tissue-Tek^®^, O.C.T. Compound (Sakura Finetek, 4583, CA, United States) and frozen in dry ice-cooled isopentane. The forebrains and brainstems were sectioned in the coronal plane and spinal cords were sectioned in the horizontal plane at 14 μm thick and collected on Superfrost Plus Microscope Slides (Fisher Scientific, 12-550-15, PA, United States).

Slides were washed in 1XPBS pH 7.4 and incubated with primary antibody, rabbit anti-active Caspase-3 (cCasp-3)(1:400, BD Biosciences, cat.# 559565, RRID:397274). The next day, slides were incubated for 1 h at room temperature with a donkey anti-rabbit IgG tagged with Alexa Fluor 488 (Thermo Fisher Scientific, cat# A21206, RRID:AB_2535792). Prior to coverslipping, nuclei were stained with bisbenzimide (Hoechst 33258; Sigma-Aldrich Inc., B1155, MO, United States).

For Western Blot analysis, tissue samples were lysed in immunoprecipitation buffer (25 mM Tris, pH 7.4, 150 mM NaCl, 1 mM CaCl, 1% Triton X-100) (Sigma, 93426, MO, United States), containing the following protease inhibitors: 200 μg/ml phenylmethylsulfonyl fluoride (Sigma, P7626, MO, United States), 1 μg/ml aprotinin (Sigma, A-6103, MO, United States) 1 μg/ml leupeptin (Sigma, L-2882, MO, United States), 1 mM dithiothreitol (DTT; Sigma, 43816, MO, United States). Protein concentration was determined with the BioRad Protein Assay reagent (500-0006, CA, United States) following manufacturer’s instructions. Proteins were run on a 10% SDS-PAGE gel with Bio-Rad Precision Plus Protein^TM^ Standards Kaleidoscope^TM^ (#161-0375 Rev B, CA, United States) molecular weight ladder. Protein were transferred to a 0.2 μm nitrocellulose membrane (Bio-Rad, 1620112, CA, United States). Immunoblotting was performed overnight with antibodies to Mcl-1 [(1:1,000); Rockland, 600-401-394, RRID:AB_2266446], anti-β-Actin [(1:2,500), Sigma, A5316, RRID:AB_476743] or anti-glyceraldehyde-3-phosphate dehydrogenase (GAPDH) (1:2,500, Cell Signaling Technology, 5714, RRID:AB_10622025) followed by the appropriate HRP-conjugated secondary antibody either Goat anti-Rabbit IgG HRP conjugate (1:2,000, BioRad, cat# 1706515, RRID:AB_11125142) or Goat anti-mouse IgG HRP conjugate (1:2,000, BioRad, cat# 1706516, RRID:AB_11125547). Blots were developed with the Western Lightning Plus-ECL Enhanced Chemiluminescence Kit (Perkin Elmer Labs Inc., 02118) according to manufacturer’s instructions.

### Microscopy, Cell Counting and Statistical Analysis

Immunostained tissues were imaged using a Zeiss AxioImager Z.1 microscope (Carl Zeiss Microscopy, Jenna, Germany) with a Colibri LED light source (Carl Zeiss Microscopy, Jenna, Germany). Images were acquired with a Zeiss AxioCam MRm camera (Carl Zeiss Microscopy, Jenna, Germany) using Zeiss AxioVision v4.8 software (Carl Zeiss Microscopy, Jenna, Germany).

Cell counts were completed using ImageJ software^1^. For counts of cCasp-3+ cells in forebrain or ventral spinal cord sections, a 125 μm × 125 μm box was placed within the lateral ganglionic eminence and the ventral-medial lumbar spinal cord, respectively. Cells were counted as cCasp-3 positive (cCasp-3 +) if they had positive immunostaining for cCasp-3 and a completely condensed nucleus, as visualized with Hoechst stain. For both brain and spinal cord counts, three representative sections were counted per embryo.

One-way ANOVA was used to compare the percent of apoptotic cells and the total number of Hoechst nuclei across the genotypes with significance assessed at α < *0.05*. Tukey’s *post hoc* analysis was performed when main effects were detected with significance assessed at *p* < *0.05*. All statistical analysis was completed using GraphPad PrismV (GraphPad Software, Inc., CA, United States).

## Results

**FIGURE 1:**
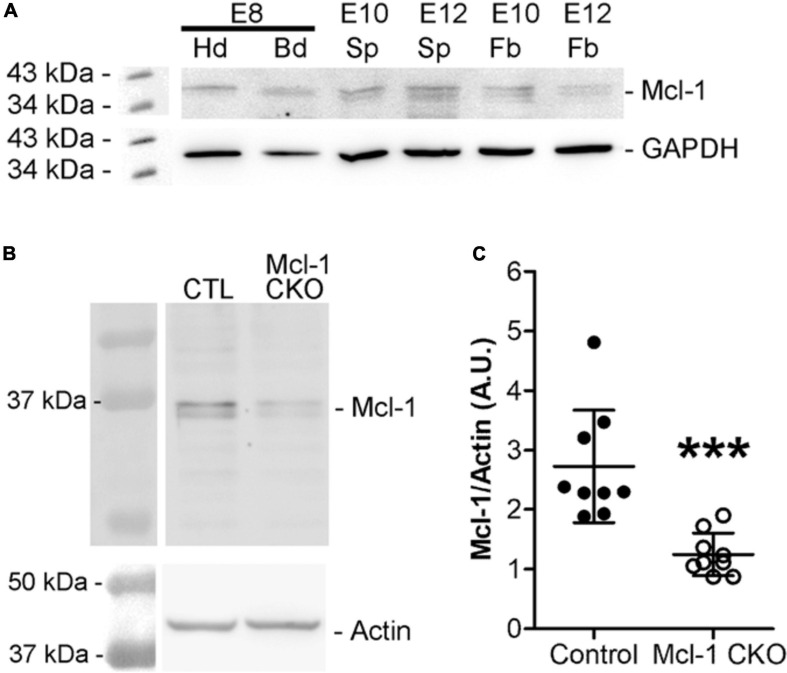
Mcl-1 protein expression is observed at E8. **(A)** Western blots for Mcl-1 in C57BL/6J wild-type E8 embryonic heads compared to E10 and E12 forebrain expression (*n* = 2 blots). Each E8 head sample contained one pooled litter. Each E10 sample contained three pooled nervous system samples and each E12 sample contained two pooled nervous system samples. GAPDH was used as a loading control. Hd, head, Bd, body, Sp, spinal cord, and Fb, forebrain **(B)** Western analysis of Mcl-1 protein expression in developing nervous system of E10 Mcl-1 CKO and littermate controls. **(C)** Densitometry analysis of Mcl-1 protein levels. Mcl-1 protein levels (A.U.) were normalized to actin (*n* = 9 samples/genotype) ***p < *0.001*, error bars represent ± SD.

To determine the anti-apoptotic role of Mcl-1 in mammalian developmental neurogenesis, we first examined the onset of Mcl-1 expression. Western blot analysis of Mcl-1 protein revealed that Mcl-1 is expressed in the head (Hd) and body (Bd) of the developing embryo as early as embryonic day 8 (E8) (Figure 1A). Mcl-1 protein is detected in neuronal tissue of the forebrain (Fb) and spinal cords (Sp) of E10 and E12 embryos. In the Mcl-1 CKO mice, a 50% reduction in Mcl-1 protein expression is observed within the nervous system by E10 (Figures 1B,C). By E12, Mcl-1 protein expression in the MKO nervous system is further reduced to ∼10% of wild type littermates (Fogarty et al., 2019).

Mcl-1 is required for neural precursor cell survival (Arbour et al., 2008; Malone et al., 2012); however, it is not clear at what point in nervous system development Mcl-1 becomes necessary for survival. To address this question, we determined the onset of apoptosis in Mcl-1 CKO embryos. We looked for the onset of apoptosis at each level of the developing nervous system: the forebrain, brainstem and spinal cord from E9 to E11. In the developing forebrain, NPC are dividing rapidly to expand their numbers at E9 just prior to the start of neurogenesis at E10, and by E11 neurogenesis is in progress (Takahashi et al., 1996). In the spinal cord, neurogenesis begins earlier as there is less of an expansion of NPC numbers. Apoptotic cells were identified by two criteria: immunopositivity for active Caspase-3 (cCasp-3) and nuclear condensation with Hoechst staining.

**FIGURE 2:**
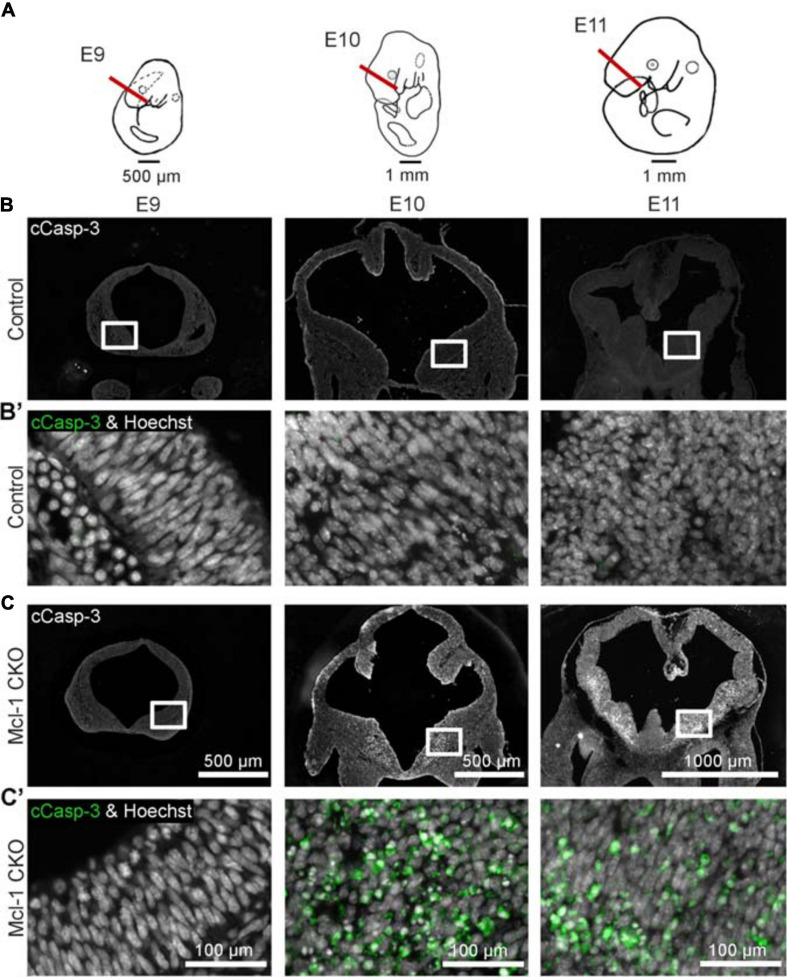
Conditional deletion of Mcl-1 results in apoptosis beginning at E10 in the forebrain. **(A)** Line drawings of E9-E11 embryos demonstrating where coronal sections from developing forebrain were collected. **(B)** Representative images of coronal sections through the forebrains of CTL embryos at E9 (*n* = 3), E10 (*n* = 2) and E11 (*n* = 4) immunostained for cCasp-3 (white). **(B′)** Higher magnification images of boxed areas in B double-labeled for cCasp-3 (green) and Hoechst (white). **(C)** Representative images of coronal sections through the forebrains of Mcl-1 CKO embryos at E9 (*n* = 3), E10 (*n* = 2) and E11 (*n* = 4) immunostained for cCasp-3 (white). **(C′)** Higher magnification images of boxed areas in C double-labeled for cCasp-3 (green) and Hoechst (white). Arrows indicate apoptotic cells.

Apoptosis was examined in coronal sections through the forebrain of Mcl-1 CKO and littermate controls at each time point (Figure 2). At E9, the developing forebrain in both control and Mcl-1 CKO embryos appeared healthy with no evidence of apoptotic cells (Figures 2B–C′). By E10, numerous apoptotic cells were observed in forebrains of Mcl-1 CKO embryos with the highest concentrations of apoptotic cells along the ventral telencephalon (Figures 2C,C′). Apoptotic cells were spread across both medial and lateral ganglionic eminences and in the dorsal cortex, small clusters were observed by E11 (Figures 2C,C′). In contrast, few to no apoptotic cells were observed in the developing forebrains of control littermates from E9 to E11 (Figures 2B,B′). This shows that in the Mcl-1 CKO, apoptosis begins at E10 in the developing forebrain starting in the ventral telencephalon and spreads laterally and dorsally.

**FIGURE 3:**
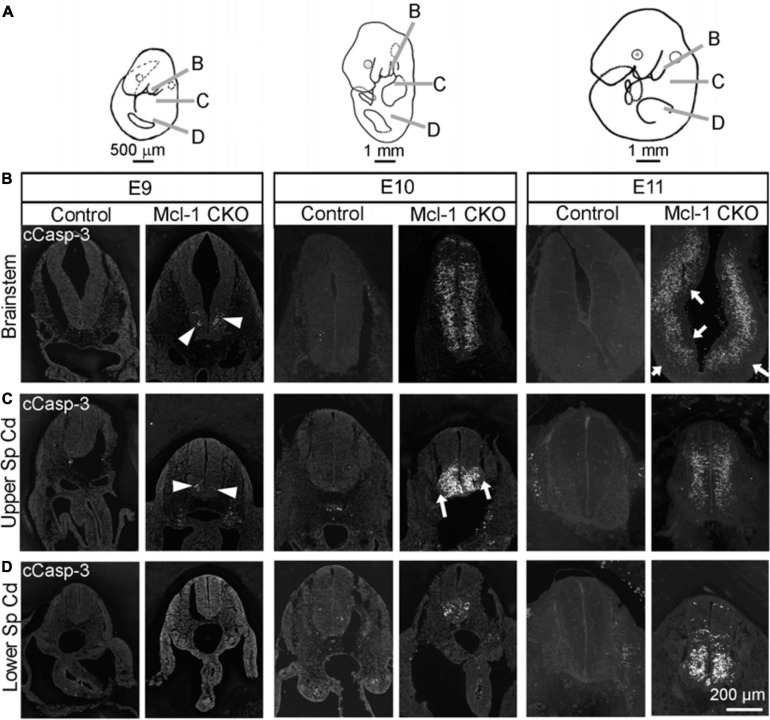
Apoptosis spreads across to axes in the brainstem and spinal cord of Mcl-1 CKO embryos. **(A)** Line drawings of embryos demonstrating the location of sections collected from the brainstem and spinal cord. Immunohistochemistry for cCasp-3 on sections from the **(B)** brainstem, **(C)** upper spinal cord, and **(D)** lower spinal cord of wild type (Control) and Mcl-1 CKO embryos from E9 to E11 (E9, *n* = 3; E10, *n* = 2; E11, *n* = 4). Arrowheads point to clusters of apoptotic cells. Arrows indicate lateral and medial regions not immuno-positive for cCasp-3.

Previous studies have focused primarily on the role of Mcl-1 in NPC within the developing brain while less is known of Mcl-1’s role in the brainstem and spinal cord. Here we examined whether apoptosis followed the same chronological pattern in the brainstem and spinal cord as in the forebrain of Mcl-1 CKO embryos. In contrast to the forebrain, small clusters of apoptotic cells first appeared in the ventral brainstem at E9 (arrowheads in Figure 3B). By E10, apoptosis had spread across the ventral to dorsal aspect of the brainstem. Apoptosis was extensive throughout the brainstem at E11, with the exception of the more lateral regions of the brainstem and a thin region lining the ventricular surface that appeared to be spared (arrows in Figure 3B). Similar to the brainstem, apoptotic cells were first observed in the ventral portion of the upper spinal cord at E9 (arrowheads in Figure 3C). By E10, apoptosis had expanded from a few cells to covering the ventral third of the cord excluding the ventral lateral horns (arrows in Figure 3D). By E11, apoptosis had spread from the ventral to all but the most dorsal region of the upper spinal cord. In contrast to the brainstem and upper spinal cord, apoptosis was initiated 1 day later at E10 in the lumbar spinal cord; however, the first few apoptotic cells were also observed in the ventral spinal cord (Figure 3C). By E11, apoptosis had spread into the dorsal spinal cord. Taken together, these results show that in the Mcl-1 CKO embryo, apoptosis spreads in two directions. Apoptosis begins rostrally in the brainstem and spreads caudally down the spinal cord from E9 to E11, and within each level, apoptosis begins in the ventral portion and spreads into the dorsal region.

To understand the anti-apoptotic role of Mcl-1 in the developing nervous system, we sought to identify the mechanism by which Mcl-1 promotes NPC survival. Mcl-1 binds and inhibits pro-apoptotic Bcl-2 effector proteins, Bax and Bak with different affinities (Chen et al., 2005). Although Mcl-1 has a stronger affinity for Bak, the role of Bak in the developing nervous system is minimal and only observed when co-deleted with Bax (Lindsten et al., 2000). In contrast to Bak, Bax has a prominent role in nervous system development including regulating the size of the neuronal population (Knudson et al., 1995; Deckwerth et al., 1996). We therefore questioned whether Bax also has a pro-apoptotic role in the proliferating NPC population. If true, Mcl-1 may promote NPC survival by inhibiting Bax activation. To address this question, we crossed the Mcl-1 CKO mice with the Bax null mouse line (Knudson et al., 1995) and examined whether the loss of Bax rescued NPC from apoptosis in the Mcl-1 CKO/Bax null embryos.

**FIGURE 4:**
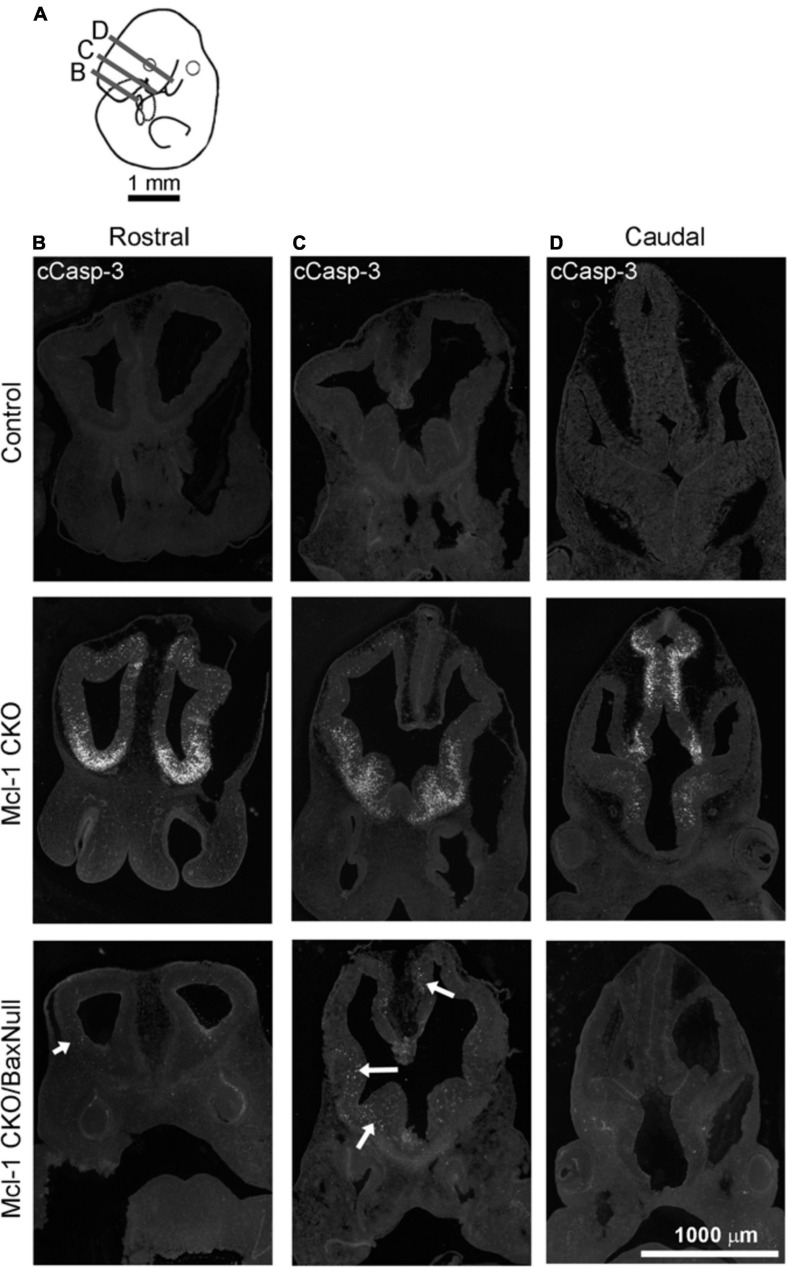
NPC are rescued from apoptotic cell death throughout the developing forebrain of E11 Mcl-1 CKO embryos when Bax is co-deleted. **(A)** Line drawing of E11 embryo indicating location of coronal sections collected through the rostral to caudal extent of the forebrain. Representative coronal sections immunostained for cCasp-3 taken from the **(B)** rostral, **(C)** middle, and **(D)** caudal portion of forebrains from wild type (Control) (*n* = 4), Mcl-1 CKO (*n* = 4) and Mcl-1 CKO/BaxNull (*n* = 4) embryos. Arrows indicate regions where there are occasional apoptotic cells in the forebrains of Mcl-1 CKO/Bax null embryos.

**FIGURE 5:**
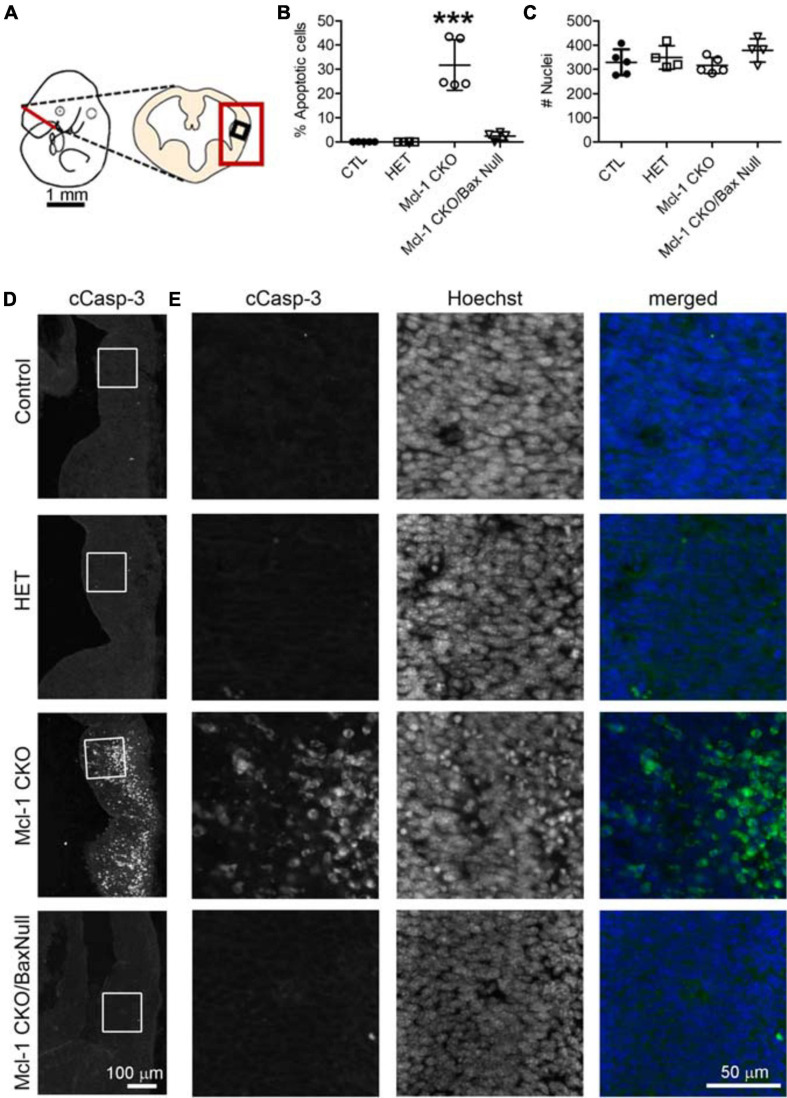
Co-deletion of anti-apoptotic Mcl-1 and pro-apoptotic Bax rescued NPC from apoptosis at E11 in the ventral forebrain. **(A)** Line diagram of an E11 embryo showing a coronal section through the forebrain at the level of the lateral ganglionic eminence. Large red box depicts where representative images were taken in D and small black box identifies the area where counts were performed in E. **(B)** The number of cCasp-3 + cells were quantified within a 125 μm × 125 μm area (boxed area in D, E) and represented as a percentage of the total number of Hoechst + nuclei. There were significantly more apoptotic cells in the Mcl-1 CKO forebrains and this was largely rescued in the Mcl-1 CKO/Bax null forebrain. **(C)** A comparison of the total number of Hoechst + nuclei revealed no significant differences across the genotypes. A one-way ANOVA was performed on the means followed by Tukey’s multiple comparison test. CTL (*n* = 5), Mcl-1 CKO (*n* = 5), Mcl-1 CKO/BaxNull (*n* = 4). **(D)** Representative sections through the ventral forebrain of E11 control, Mcl-1 CKO and Mcl-1 CKO/BaxNull embryos. **(E)** Higher magnification of boxed areas in D showing individual panels for cCasp-3 immunostaining, Hoechst and merged (cCasp-3 = green, Hoechst = blue). ***p < *0.001*, error bars represent ± SD.

Apoptosis was examined in coronal sections throughout the rostral to caudal extent of the developing forebrain at E11 (Figure 4A). Numerous apoptotic cells were observed in the ventral forebrains of Mcl-1 CKO embryos with the majority of cells located in the medial and lateral ganglionic eminences (Figures 4B–D). In the Mcl-1 CKO/BaxNull forebrain, only a scattered few apoptotic cells were observed throughout the forebrain (arrows in Figures 4B,C). To determine the extent at which co-deletion of Bax rescues apoptotic NPC in the Mcl-1 CKO, we compared the number of apoptotic cells within a 125 μm × 125 μm boxed area in the lateral ganglionic eminences of Ctl (*n* = 5), Mcl-1 Het (*n* = 4), Mcl-1 CKO (*n* = 5) and Mcl-1 CKO/BaxNull embryos (*n* = 4) (Figures 5A,D,E). In both control littermates and Mcl-1 Het embryos there were few to no apoptotic cells observed (Figures 5B,D,E). In the Mcl-1 CKO embryos ∼31.7 ± 10.4% of cells were apoptotic whereas, in the Mcl-1 CKO/BaxNull embryos, only 2.4 ± 1.7% of cells were apoptotic. A one-way ANOVA revealed a main effect of genotype on the percent of apoptotic cells [*F*(3,17) = 36.45, *p* < 0.0001] (Figure 5B). Tukey’s multiple comparison *post hoc* test showed that Mcl-1 CKO had significantly more apoptotic cells than all other genotypes (*p* < *0.001*) and that there was no significant difference in the percent of apoptotic cells in the Mcl-1 CKO/BaxNull compared to control and Mcl-1 Het. To test whether the density of cells within our counting area was comparable across genotypes, a one-way ANOVA was performed on the mean number of nuclei per boxed area. No significant differences were found across the different genotypes [*F*(3,17) = 1.498, *p* = 0.2584] (Figure 5C).

**FIGURE 6:**
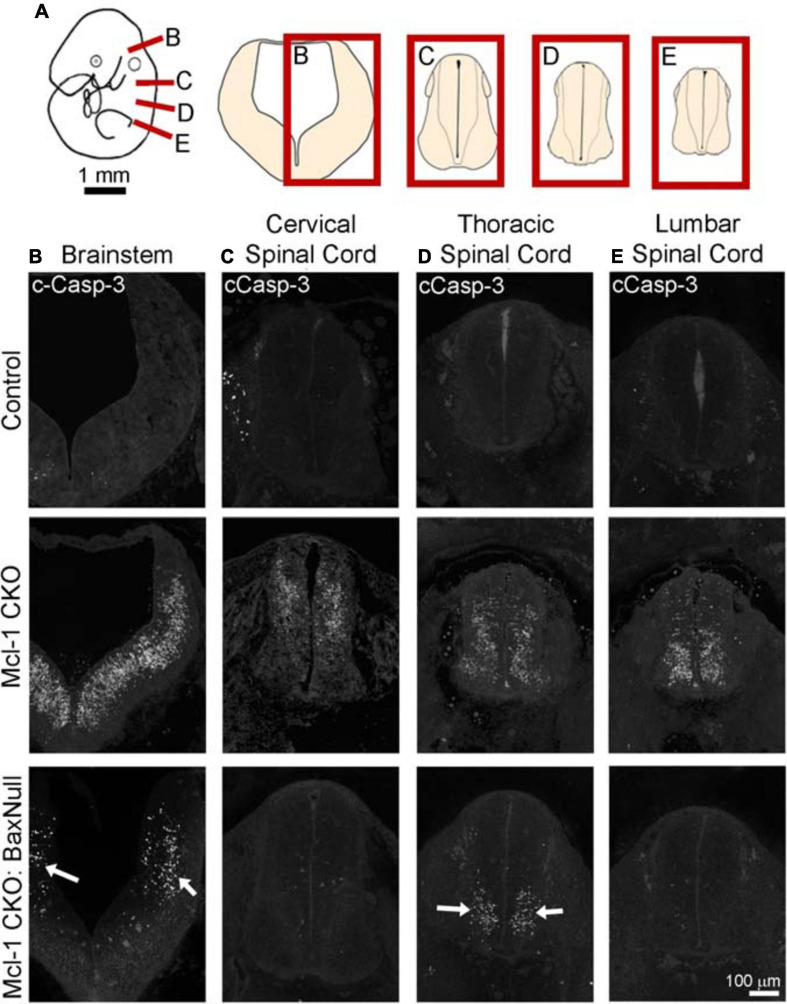
Co-deletion of Mcl-1 and Bax does not rescue all NPC from apoptotic cell death in the developing nervous system at E11. **(A)** Diagram of an E11 embryo showing the levels of brainstem and spinal cord. Representative sections of cCasp-3 immunohistochemistry (white) at the level of the **(B)** brainstem, **(C)** cervical spinal cord, **(D)** thoracic spinal cord, and **(E)** lumbar spinal cord of CTL (*n* = 4), Mcl-1 CKO (*n* = 4) and Mcl-1 CKO/BaxNull (*n* = 3). Arrows point to apoptotic cells in Mcl-1 CKO/BaxNull embryos.

**FIGURE 7:**
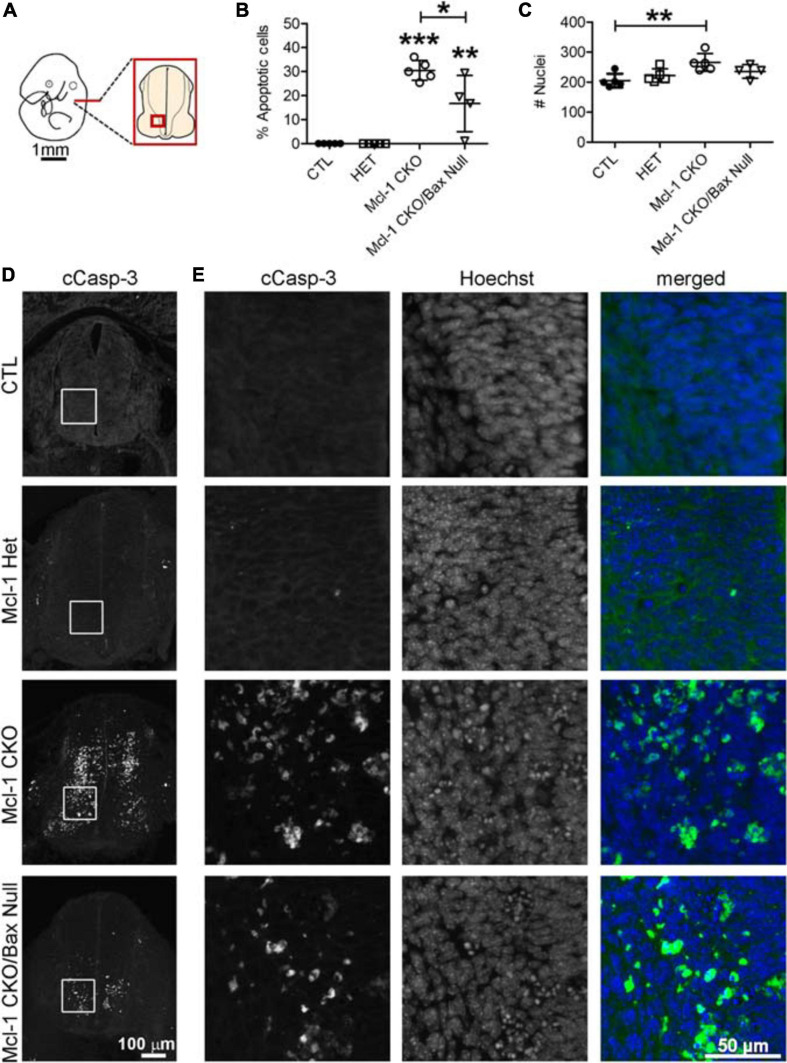
Incomplete rescue of NPC cell death in the developing spinal cord of Mcl-1 CKO/BaxNull embryos. **(A)** Line diagram of an E11 embryo depicting the location of spinal cord sections. **(B)** The number of cCasp-3 + cells were quantified within a 125 μm × 125 μm area (boxed area in D, E) and represented as a percentage of the total number of Hoechst + nuclei. Co-deletion of Bax in the Mcl-1 CKO/BaxNull embryo rescued 50% of NPC from apoptosis in the ventral spinal cord. **(C)** A comparison of the total number of Hoechst + nuclei revealed more cells in the Mcl-1 CKO, which may be due to quantification of apoptotic bodies versus healthy nuclei. A one-way ANOVA was performed on the means followed by Tukey’s multiple comparison test. **(D)** Representative sections through the ventral spinal cords of E11 controls Mcl-1 Het, Mcl-1 CKO and Mcl-1 CKO/BaxNull embryos. **(E)** Higher magnification of boxed counting areas in D showing individual panels for cCasp-3 immunostaining, Hoechst and merged (cCasp-3 = green, Hoechst = blue). *p < *0.05*, **p < *0.01*, ***p < *0.001*, and error bars represent ± SD.

To determine whether Bax also rescues apoptotic NPC throughout the entire nervous system in the Mcl-1 CKO, we examined the brainstem and cervical, thoracic and lumbar levels of the spinal cords (Figure 6A). In E11 control littermates, only a few apoptotic cells were observed in the ventral brainstem (Figure 6B). In the Mcl-1 CKO embryo, apoptosis was widespread throughout the brainstem, and extended across the ventral to dorsal spinal cord at each level (Figures 6B–E). In contrast to the results in the forebrain, NPC in the Mcl-1 CKO did not appear to be completely rescued in the brainstem and spinal cord with co-deletion of Bax. Clusters of apoptotic cells were observed in the dorsomedial region of the brainstem and in the ventral thoracic spinal cord of the Mcl-1 CKO/BaxNull embryo (arrows in Figures 6B,D). NPC were rescued at the cervical and lumbar levels of the spinal cord. To determine the extent at which co-deletion of Bax rescues apoptotic cells in the Mcl-1 CKO spinal cord, we compared the number of apoptotic cells within a 125 μm × 125 μm boxed area in the ventral thoracic spinal cords of Ctl (*n* = 5), Mcl-1 Het (*n* = 5), Mcl-1 CKO (*n* = 5) and Mcl-1 CKO/BaxNull embryos (*n* = 4) (Figures 7A,D,E). Similar to the apoptosis counts in the forebrain, there is little to no apoptosis in the spinal cords of littermate control or Mcl-1 HET embryos (Figures 7B,D,E). In the Mcl-1 CKO embryos 30.5 ± 4.1% of cells were apoptotic whereas, 16.7 ± 11.7% of cells were apoptotic in the Mcl-1 CKO/BaxNull embryos, demonstrating a 50% rescue with Bax co-deletion. A one-way ANOVA revealed a main effect of genotype on the percent of apoptotic cells [*F*(3,18) = 33.64, *p* < 0.001] (Figure 7B). Tukey’s multiple comparison *post hoc* test showed that the Mcl-1 CKO (*p* < *0.0001)* and Mcl-1 CKO/BaxNull (*p* < *0.01*) spinal cords had significantly more apoptotic cells than control and Mcl-1 Het. Furthermore, Mcl-1 CKO/BaxNull had significantly fewer apoptotic cells than Mcl-1 CKO (*p* < *0.05*). To test whether the density of cells within our counting area was comparable across genotypes, a one-way ANOVA was performed on the mean number of nuclei per boxed area. A main effect of genotype was detected [*F*(3,18) = 5.29, *p* < 0.05]. The follow-up Tukey’s multiple comparison *post hoc* revealed that there were significantly more nuclei within the Mcl-1 CKO counting area than in the littermate controls (Figure 7C). As the total nuclei counts include both healthy and apoptotic nuclei, difficulties arise with apoptotic nuclei fragmenting which may slightly skew the overall nuclei counts. These results show that Bax co-deletion rescued 50% of cells from apoptosis in spinal cord of the Mcl-1 CKO in contrast to the almost complete rescue in the forebrain.

Co-deletion of Bax and Mcl-1 rescued most NPC from apoptotic cell death throughout the developing nervous system. Despite this rescue, Mcl-1 CKO/BaxNull embryos did not survive beyond E12.5 (Table 1). In surprising contrast, Mcl-1 CKO embryos are lethal at E15.5 and Bax Null mice are viable and healthy (Knudson et al., 1995; Arbour et al., 2008). We questioned whether the conditional deletion of Bax only in the nervous system would rescue the embryonic lethality. Transgenic mice carrying floxed Bax alleles were crossed with Mcl-1 CKO/BaxNull mouse line and viable offspring were born and survived up to postnatal day 16 (Table 2). This demonstrates that co-deletion of Bax rescues both cell death within the CNS as well as the embryonic lethality.

**TABLE 1:** Survival of Mcl-1 CKO/BaxNull embryos.

**Mcl-1 CKO/BaxNull**	**Embryonic time point**		
	**E11**	**E12**	**E13**
Number of litters	17	3	16
Number of embryos	173	22	136
Number of knock-outs (%)	13 (7.51%)	1 (4.55%)	1 (0.74%)
Predicted number with Mendelian ratio (%)	11 (6.25%)	1 (6.25%)	7 (5.31%)

**TABLE 2:** Survival of Mcl-1 CKO/BaxNull/BaxFloxed embryos.

**Mcl-1 CKO/BaxNull: BaxFloxed**	**Embryonic and postnatal time points**					
	**E11**	**E13**	**E15**	**E18**	**P2**	**P16**
Number of litters	9	1	7	1	3	1
Number of embryos	86	9	66	7	27	9
Number of knock-outs (%)	9 (10.47%)	1 (11.11%)	16 (24.24%)	1 (14.29%)	3 (11.11%)	2 (22.22%)
Predicted number with Mendelian ratio (%)	8 (9.08%)	2 (25%)	12 (18.94%)	2 (25%)	5 (17.13%)	2 (25%)

## Discussion

This study investigated the anti-apoptotic role of Mcl-1 in mammalian neurogenesis. There are two main findings: first, Nestin:Cre mediated deletion of Mcl-1 results in apoptosis throughout the spinal cord, brainstem, and forebrain and coincides with the initiation of neurogenesis. Second, Mcl-1’s anti-apoptotic function in the developing nervous system is to inhibit Bax activation. Although co-deletion of Mcl-1 and Bax rescued the majority of cells from apoptosis throughout the nervous system, the rescue was incomplete in the brainstem and ventral thoracic spinal cord, demonstrating that Bax is not the sole pro-apoptotic target of Mcl-1 in the developing nervous system.

### Mcl-1 Is Required for Developmental Neurogenesis

Mcl-1 has a role in early embryonic development as germline deletion of *mcl-1* is embryonic lethal at E3.5 (Rinkenberger et al., 2000). Mcl-1 is expressed in the early murine embryo, with expression peaking at E5 and then decreasing slightly by E6.5 (Rinkenberger et al., 2000). In the developing nervous system, *mcl-1* mRNA was detected at E10 in the spinal cord and at E11 in the forebrain; however, earlier time points were not assessed (Arbour et al., 2008; Fogarty et al., 2019). As formation of the murine neural tube from the neural plate begins at E8, we investigated this time point to determine whether Mcl-1 was expressed during patterning of the nervous system (Copp et al., 2003). Mcl-1 protein is expressed in the murine embryonic head at E8 as demonstrated by western blot. The small size of the E8 mouse embryo hindered a clean dissection of neural tissue from non-neural tissue although the majority of the tissue sample was neural tissue. Mcl-1 therefore is expressed early in nervous system development.

Mcl-1 is not required for patterning of the nervous system. We have previously shown that the early stages of nervous system development including neural tube formation are not affected in either the Mcl-1 CKO or the double Mcl-1 and Bclx CKO embryo (Fogarty et al., 2019). Here, we show that this is because apoptosis only begins at E9 in the spinal cord and E10 in the forebrain after early patterning of the nervous system is completed and coincides with the onset of neurogenesis. Our findings are consistent with knockout mouse studies of the pro-apoptotic Bcl-2 family members where deletion of two or all three of the multi BH3 domain pro-apoptotic proteins, Bax^–/–^:Bak^–/–^ or Bax^–/–^:Bak^–/–^:Bok^–/–^ revealed that apoptosis is not required for morphogenesis of the developing nervous system, but rather for regulating the size of neuronal populations (Lindsten et al., 2000, 2003; Ke et al., 2018).

Mcl-1’s anti-apoptotic role is linked to NPC differentiation. Mcl-1 was initially identified in a screen for genes that are upregulated during myeloid cell differentiation (Kozopas et al., 1993). Germline deletion of *mcl-1* is peri-implantation lethal due to a failure of trophectoderm differentiation (Rinkenberger et al., 2000). We have previously shown that apoptotic cells in the Mcl-1 CKO double label with antibodies to Nestin, a neural precursor marker, or doublecortin, a neuroblast marker or with βIII tubulin-1, an immature neuron marker (Arbour et al., 2008). This demonstrates that NPC die during the process of differentiation from proliferating cell to immature neuron. In this study, we show that apoptosis in the Mcl-1 CKO begins earlier in the developing brainstem and rostral spinal cord than in the ventral forebrain and then spreads across the nervous system. This differential timing of apoptosis corresponds with timing and spread of neurogenesis in each region. Neurogenesis begins at E9.5 in the spinal cord (Alaynick et al., 2011). In the forebrain, expansion of the NPC pool occurs from E9–E10 preceding the onset of neurogenesis at E11 (Angevine and Sidman, 1961; Takahashi et al., 1995). In each region of the nervous system, apoptosis started in the ventral cell populations and over time spread across two axes, from ventral to dorsal as well as from rostral to caudal. This progression closely mirrors the pattern of differentiation in the developing nervous system. Differentiation in the spinal cord also begins ventrally and progresses dorsally (McConnell, 1981). Furthermore, rostral signaling by retinoic acid released from the somatic mesoderm causes cells of the developing neural tube to differentiate, whereas caudal Wnt and Fgf signaling prevents differentiation. This results in a progression of differentiation that begins rostrally and progresses caudally over time (Gouti et al., 2015). The similarity between the progression of apoptosis in the Mcl-1 CKO and the progression of differentiation in the developing nervous system suggests that NPC are dependent on Mcl-1 for cell survival during developmental neurogenesis.

### Mcl-1 Exerts Its Pro-survival Function Through Inhibition of Bax Activation in NPC

Mcl-1 blocks the initiation of apoptosis by binding and inhibiting the activity of BH3-only proteins and the multi BH-domain pro-apoptotic proteins, Bax and Bak (Czabotar et al., 2014). As Mcl-1 has multiple pro-apoptotic targets, we focused on Bax, as it is the dominant pro-apoptotic protein expressed in the developing nervous system (Krajewski et al., 1994). Germline deletion of Bax rescues neurons from developmental death resulting in a 24–35% increase in neuronal populations (Deckwerth et al., 1996; White et al., 1998). In contrast, Bak null mice do not appear to have a CNS phenotype (Lindsten et al., 2000). Germline deletion of both Bax and Bak worsens the severity of the Bax null phenotype and expands the populations of NPC, immature neurons and glial cells, indicating they both have a role in regulating the NPC population (Lindsten et al., 2000). Our data and previous data show that co-deletion of Mcl-1 and Bax rescues the majority of apoptotic cells in the nervous system of Mcl-1 CKO embryos (Fogarty et al., 2019). However, a thorough rostral to caudal assessment of the entire nervous system at E11 revealed only a partial reduction in apoptosis. Clusters of apoptotic cells remained in the dorsal brainstem and ventral thoracic spinal cord of Mcl-1 CKO:Bax Null embryos demonstrating that Bax deletion alone is insufficient to prevent apoptosis entirely in the Mcl-1 CKO embryos. These findings demonstrate that Mcl-1 promotes cell survival primarily by blocking the pro-apoptotic activity of Bax during early developmental neurogenesis. The incomplete rescue indicates that Bax is not the sole pro-apoptotic target of Mcl-1 in the developing nervous system. Further investigations are necessary to identify additional pro-apoptotic targets of Mcl-1. Interestingly, although germline deletion of Bax rescued the majority of cells from apoptosis, it resulted in earlier embryonic lethality at E12 versus E15.5 in the Mcl-1 CKO embryo. In contrast, conditional deletion of Bax did rescue NPC apoptosis and embryonic lethality.

### The Changing Role of Mcl-1 During Nervous System Development

The role of Mcl-1 in nervous system development has been examined with a variety of conditional knockout mice, each demonstrating unique roles for Mcl-1 in different cell populations. Foxg1-Cre and Nestin-Cre mediated deletion of Mcl-1 results in loss of Mcl-1 in the NPC population early in development of the forebrain and central nervous system, respectively (Akagi et al., 2001; Berube et al., 2005; Arbour et al., 2008). Both conditional knockouts resulted in Caspase-3 activation and widespread NPC apoptosis (Arbour et al., 2008). Similarly, electroporation or transfection of the Nestin-Cre plasmid into adult NPC carrying floxed Mcl-1 alleles (Mcl-1^*flox/flox*^) resulted in a 50% loss of NPC by apoptosis (Malone et al., 2012). This demonstrates that Mcl-1 is required for the survival of NPC from the onset of neurogenesis in the developing nervous system and into the adult brain. Further studies including the current showed that Mcl-1 maintains NPC survival during the stages of neurogenesis as cells exit the cell cycle to differentiate into neurons (Arbour et al., 2008; Fogarty et al., 2019). Nestin-Cre mediated deletion of both Mcl-1 and Bcl-xL revealed that Mcl-1 is the main anti-apoptotic regulator of NPC survival with Bcl-xL having a partially supportive role. Once NPC exit the cell cycle and become post-mitotic immature neurons, the roles change with Bcl-xL being the main anti-apoptotic regulator while Mcl-1’s role is greatly diminished (Fogarty et al., 2019). In contrast to NPC in the embryonic CNS, committed neural progenitors and cerebellar ganglion neurons (CGN) in the postnatal cerebellum are not dependent on Mcl-1 for survival (Crowther et al., 2013; Veleta et al., 2020). Math1-Cre mediated deletion of Mcl-1 does not result in apoptosis of cerebellar neural progenitors and CGN, rather conditional deletion of Bcl-xL does (Crowther et al., 2013). Math1-Cre mediated co-deletion of Mcl-1 and Bcl-xL reveals that Mcl-1 functions in a subsidiary role to Bcl-xL by exacerbating the loss of Bcl-xL (Veleta et al., 2020). As post mitotic neurons differentiate and mature they become more resistant to apoptosis and the role of Mcl-1 also changes (Kole et al., 2013; Annis et al., 2016). CamKII-Cre mediated deletion of Mcl-1 in cortical neurons results in autophagic cell death demonstrating that Mcl-1 functions in an anti-autophagic role in post mitotic neurons (Germain et al., 2011). Taken together, these studies demonstrate the roles of Mcl-1 are dynamic, changing through development and with different cell populations.

In summary, we show that Nestin-Cre mediated deletion of Mcl-1 results in apoptotic NPC cell death in the embryonic spinal cord, brainstem and forebrain demonstrating that NPC throughout the developing nervous system are dependent on Mcl-1 for survival. Apoptosis coincides with the onset of neurogenesis in each region of the developing nervous system indicating that Mcl-1 is required during cell differentiation but not prior. Co-deletion of Mcl-1 and Bax rescued the vast majority of cells from apoptosis demonstrating that Mcl-1 functions primarily to inhibit pro-apoptotic Bax during developmental neurogenesis. However in the ventral thoracic spinal cord and in the dorsal brainstem, there were still a significant number of apoptotic cells, demonstrating the presence of other pro-apoptotic targets of Mcl-1. In conclusion, these findings focus the anti-apoptotic role of Mcl-1 in the embryonic nervous system to the neurogenic period.

## Data Availability Statement

The raw data supporting the conclusions of this article will be made available by the authors, without undue reservation.

## Ethics Statement

The animal study was reviewed and approved by Memorial University Animal Care Committee.

## Author Contributions

RF and JV designed the experiments. RF, SC, BG performed the experiments and gathered the data. RF and JV wrote the manuscript. JO provided the Mcl1 floxed mice, provided input on the design of the project and the final manuscript. All authors contributed to the article and approved the submitted version.

## Conflict of Interest

The authors declare that the research was conducted in the absence of any commercial or financial relationships that could be construed as a potential conflict of interest.

## Footnotes

1 http://imagej.nih.gov/ij/
